# Comparison of radiographic changes in rectangular curved short stem with thin versus thick porous coating for cementless total hip arthroplasty: a retrospective study with a propensity score matching

**DOI:** 10.1186/s13018-021-02397-3

**Published:** 2021-04-13

**Authors:** Yutaro Munakata, Yujiro Kuramitsu, Yutaka Usui, Ken Okazaki

**Affiliations:** Department of Orthopaedic Surgery, Tokyo Women’s Medical University, 8-1, Kawada-cho, Shinjuku-ku, Tokyo, 162-8666 Japan

**Keywords:** Short stems, Radiologic evaluation, Propensity score matched analysis

## Abstract

**Background:**

Radiographic changes that appear relatively quickly after fixation of cementless stem in total hip arthroplasty (THA) vary depending on the stem design and fixation style. The present study compared radiographic changes between two types of rectangular curved short stems of similar shape.

**Methods:**

This retrospective study included 118 hips that underwent primary cementless THA with an anterolateral supine approach using a rectangular, curved, short stem performed by the same surgeon between June 2015 and June 2019. Among the examined hips, 39 had a thicker porous coating stem (thicker group) and 66 had a thinner porous coating and reduced tip stem (thinner group) and at least 12-month follow-up. Radiographs taken during the final course observation were assessed. Propensity score matching was performed based on demographic data and comparisons were made using pairs of 25 hips each. Statistical analysis was performed using chi-square test and *p* values ≤ 0.05 indicated statistical significance.

**Results:**

The pattern of the radiolucent lines showed a significant difference after matching (*p* = 0.0044). A “proximal and distal” pattern was most common in the thicker group and a “distal only” pattern was most common in the thinner group. There was notable and significant difference in cortical hypertrophy in the thicker group after matching (*p* = 0.024).

**Conclusions:**

Although the two short stems were similar shapes, the short-term radiographic changes were different. The thinner group showed fewer radiographic changes than the thicker group, making it a more “silent” stem.

## Background

Total hip arthroplasty (THA), hailed as “the operation of the century,” was the most successful surgery in the last century [1]. During its development, a “short stem” was designed to obtain more physiological load transfer with a less invasive procedure to preserve bone and soft tissue [2]. Recent comparisons with standard stems have reported that short stems are considered equivalent to standard stems in clinical assessment and imaging over short periods of time [3, 4]. Koyano et al. examined 36 cases of simultaneous bilateral THA, where a short stem was used on one side and a standard stem was used on the other, over an average of 9.2 years. Their radiological findings suggested that the short stem transferred the load more proximally than the standard stem, while no difference was observed in the hip score [5]. Yu et al. reported no difference in clinical assessment and imaging in 55 hips with short stem and 58 hips with standard stem, even for patients aged ≥ 70 years, over short-term (3–4 years), and thigh pain and intraoperative fractures were less common with the short stem [6]. On the other hand, in an Italian regional registry, use of a short stem in the “standard osteotomy” group showed significantly more revisions due to pain compared with other groups [7]. According to the 2018 report by the Australian Orthopaedic Association National Joint Registry, loosening and revision rates were higher in mini stems than in conventional stems [8]. However, there were improvements in the 2020 report [9]. Therefore, controversy still exists regarding the radiological stability of short stems. One cause is that various design concepts are included within implants that are classified as short and mini stems. Furthermore, there may be differences among systems with similar shapes in terms of the surface finishing, such as the porous coating that may influence the postoperative radiographic changes.

**Fig. 1:**
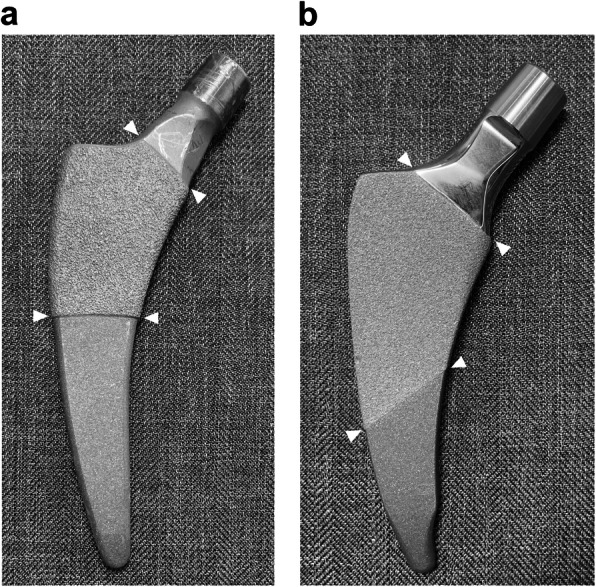
Appearance of both the groups is depicted. **a** Appearance of the thicker group. It has thicker (0.4 mm), symmetrical porous coating and stem tip is not reduced. Arrows indicate the area of the porous coating. **b** Appearance of the thinner group. It has thinner (0.2 mm), asymmetrical porous coating and stem tip is reduced. Arrows indicate the area of the porous coating

Rectangular curved short stems comprise a short stem with a rectangular (trapezoidal) cross-section [10], similar to the Zweymüller type [11] with smooth curves. An example of this type of stem is the Fitmore (ZIMMER BIOMET, Warsaw, IN, USA, Fig. 1a), which has been reported to have stable short-term and mid-term performance [4, 12–15], but the occurrence of cortical hypertrophy is reported to be common (29–71%) [12–16]. Meanwhile, there is a limited number of reports regarding the performance of the Minima (LIMA Corporate, San Daniele Del Friuli, Udine, Italy, Fig. 1b), [17, 18] rectangular curved short stem, and clinical results and radiographic changes have not been adequately examined. Although these systems have a similar shape, they differ in the shape of the tip, thickness, and extent of the porous coating. The Fitmore stem has a thicker porous coating than the Minima stem. Radiographical changes may develop relatively quickly following surgery. They are often related to stem fixation and may be associated with clinical outcomes, such as the need for early revision [19, 20].

The present study aimed to elucidate the differences in radiographic changes that appear shortly after surgery using two types of rectangular curved short stems placed with the same surgical method by the same surgeon. We performed a propensity score-matched analysis to make patient background uniform using factors that likely have an impact on the result, including gender, age, body mass index, follow-up period, and morphology of the femoral canal [21].

## Methods

The present study was reviewed by the ethics committee of our facility (approval number 5280, August 13, 2019). Informed consent was obtained using an opt-out form on the center’s website. All data were handled according to the ethical standards of the Declaration of Helsinki.

The study included 124 hips that consecutively underwent primary cementless THA, which was performed with an anterolateral supine approach using a rectangular curved short stem by the same surgeon between June 2015 and June 2019. Preoperative diagnoses were hip dysplasia, avascular necrosis of the femoral head, rapidly destructive coxopathy, primary osteoarthritis of the hip, femoral neck fracture, rheumatoid arthritis, post-traumatic arthritis, and septic arthritis. The Fitmore and Minima stems were used and were selected by the surgeon based on the medial curvature of the proximal femur and lateral offset in preoperative two-dimensional templating. Fitmore has a thicker porous coating, while Minima has a thinner coating. Exclusion criteria were cases that underwent osteosynthesis for hip fracture, existing inflammatory disease, postoperative periprosthetic fracture, and cases that required additional surgery due to infection or problem with the cup side. Totals of 42 hips with Fitmore and 76 hips with Minima were included in the study. Among these, 13 hips could not be followed up for 12 months; thus, ultimately the study included 39 hips with Fitmore (thicker group) and 66 hips with Minima (thinner group). The follow-up rate was 89.0%. We collected data for age, gender, and body mass index for these cases from medical records. In order to assess the femoral canal morphology, we measured the canal flare index [22] from preoperative anteroposterior radiograph in a supine position.

### Clinical assessment

We measured the Japanese Orthopaedic Association (JOA) hip score prior to surgery and at the time of final course observation for evaluation. JOA hip score is an objective clinical score consisting of four subcategories, including pain around the hip joint, range of motion, walking ability, and activities of daily living, validated in patients with hip osteoarthritis [23].

### Radiological assessment

**Fig. 2:**
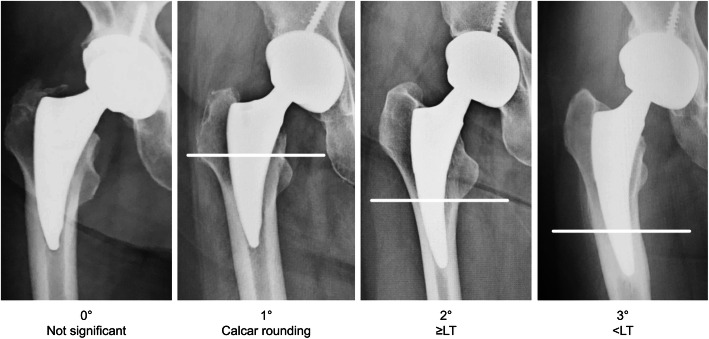
Stress shielding assessment. LT, lesser trochanter

**Fig. 3:**
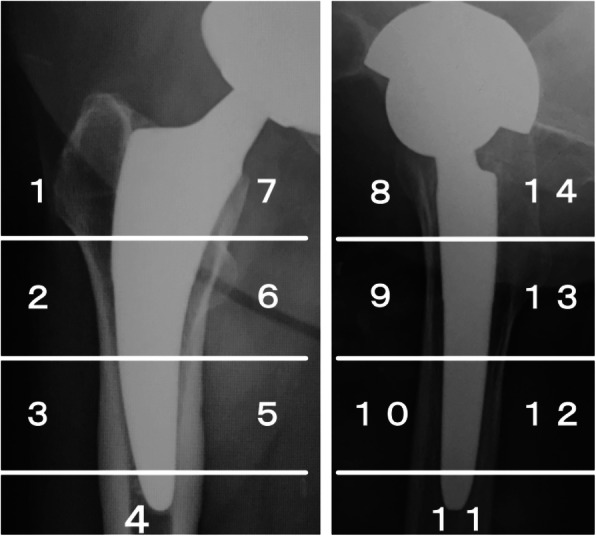
Gruen zones in anteroposterior and lateral radiograph. According to this zoning, radiolucent lines and cortical hypertrophy were evaluated

We used an anteroposterior radiograph in a supine position and Lauenstein images immediately after surgery and at the time of the final course observation to evaluate radiographic changes in the areas around the stem after surgery. For stress shielding, we modified the Engh Grading Scale [24] and made an evaluation based on anteroposterior radiograph in the supine position only. No clear bone resorption was defined as “nothing,” calcar rounding was defined as first degree, bone resorption advanced to the bottom of the lesser trochanter was defined as second degree, bone resorption advanced beyond the lesser trochanter was defined as third degree, and bone resorption advanced to the stem tip was defined as fourth degree. Since no cases were classed as fourth degree, we only assessed to third degree (Fig. 2). The radiolucent lines were assessed in two directions (anteroposterior radiograph in the supine position and Lauenstein imaging) and findings ≥ 1 mm was considered “present.” Anterior and lateral modified Gruen zones [17, 25] (Fig. 3) 1, 7, 8, and 14 were defined as “proximal,” and 3, 4, 5, 10, 11, and 12 were defined “distal,” and then classified as either “nothing,” “proximal only” (more proximal than the top of the lesser trochanter), “distal only,” or “proximal and distal.” No cases showed a radiolucent line that straddled the middle part extending from proximal to distal throughout the whole length. Cortical hypertrophy was also examined in the same two directions, and the number of confirmed modified Gruen zones [17, 25] (Fig. 3) was evaluated. Stem subsidence was only evaluated on the anteroposterior radiograph in the supine position, and subsidence of ≥ 3 mm was considered significant.

Interobserver and intraobserver reproducibility was assessed using two examiners to evaluate radiograph twice in a blinded manner in 10 randomly selected patients.

### Statistical analysis

Statistical analysis of the interobserver and intraobserver reproducibility was performed using Gauge R & R analysis. Student’s *t* test was used for continuous variables with normal distributions. Chi-square test was used for categorical variables. All *p* values ≤ 0.05 were considered statistically significant. Propensity score matching was performed to adjust covariates including gender, age, body mass index, follow-up period, and canal flare index with a caliper width of 0.2 as a logit transformation [26]. Demographic data, JOA hip score, and radiographic changes were compared between the groups before and after matching. JMP Pro 13.0.0 (SAS, Cary, NC, USA) was used for statistical analyses.

## Results

Results of the Gauge R & R analysis for evaluating interobserver and intraobserver reproducibility showed 80% consistency and a Kappa value of 0.7 for stress shielding, 100% consistency and a Kappa value of 1 for radiolucent lines, 90% consistency and a Kappa value of 0.84 for cortical hypertrophy, and 100% consistency and a Kappa value of 1 for stem subsidence. Reproducibility was acceptable in all the assessments.

The demographic data before and after matching is shown in Table 1. Before matching, there was significant difference in gender, age, and follow-up between the groups, but these differences disappeared after matching.

**Table 1:** Demographic data before and after propensity score matching. Data represent mean (standard deviation)

	Thicker group	Thinner group	*p* value
Before matching	*n* = 39	*n* = 66	
Gender (male to female)	11:28	8:58	0.064
Age, years	64.9 (13.0)	71.4 (10.1)	0.0053
Body mass index, kg/m^2^	23.0 (4.5)	23.9 (5.0)	0.36
Follow-up, months	33.4 (11.5)	23.3 (11.3)	< 0.0001
Canal flare index	3.4 (0.5)	3.5 (0.4)	0.18
After matching	*n* = 25	*n* = 25	
Gender (male to female)	4:21	3:22	1
Age, years	70.5 (12.0)	69.3 (10.4)	0.71
Body mass index, kg/m^2^	22.0 (4.4)	22.0 (4.6)	0.96
Follow-up, months	31.6 (10.8)	30.2 (12.2)	0.68
Canal flare index	3.4 (0.55)	3.4 (0.46)	0.76

JOA hip scores are shown in Table 2 as clinical results. Both groups showed notable improvements at the time of final course observation, but there was no significant difference between the groups before or after the matching.

**Table 2:** JOA hip score results. Data represent mean (standard deviation)

	Thicker group	Thinner group	*p* value
Before matching	*n* = 39	*n* = 66	
Pre-surgical	31.7 (22.7)	33.6 (16.7)	0.61
At the final follow-up	90.8 (8.0)	89.5 (7.4)	0.39
After matching	*n* = 25	*n* = 25	
Pre-surgical	27.0 (19.5)	37.4 (20.8)	0.07
At the final follow-up	90.0 (7.2)	91.4 (6.4)	0.46

**Fig. 4:**
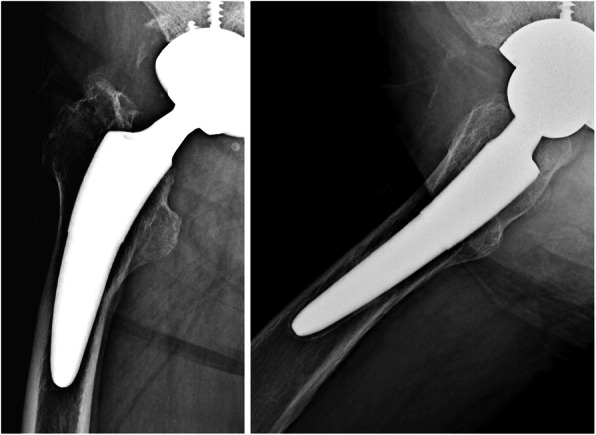
Example of “proximal and distal” pattern. It was the most common pattern for radiolucent lines in the thicker group. Gruen zones 1, 3, 4, 8, 10, 11, and 12 showed radiolucent lines with widths of 1–2 mm

**Fig. 5:**
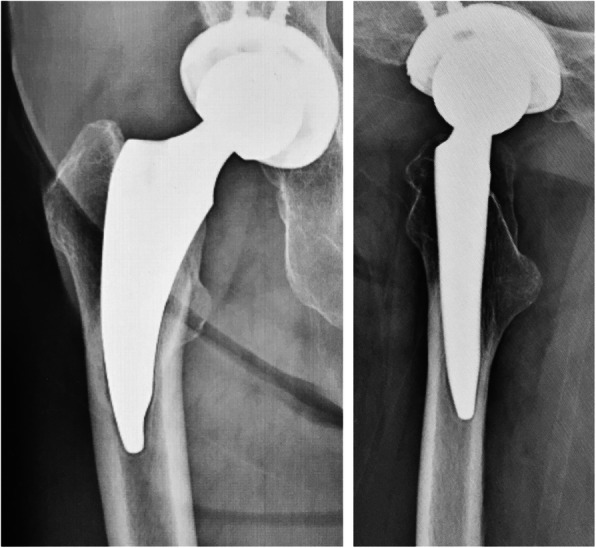
Example of “distal only” pattern, the most extensive case in the thinner group. It was the most common pattern for radiolucent lines in the thinner group. Gruen zone 3, 4, 5, 10, 11, and 12 present radiolucent lines with the width of 1–2 mm

The pre-matching and post-matching results for radiographic changes are shown in Table 3. Stress shielding showed a significant difference (*p* = 0.015) before matching, but this disappeared after matching (*p* = 0.26). However, second degree was most common in thicker group (9 hips) and first degree was most common in thinner group (14 hips). The patterns of radiolucent lines showed a significant difference before matching (*p* < 0.0001) and after matching (*p* = 0.0044). In the thicker group, “proximal and distal” was most common and was seen in 10 hips (Fig. 4), while “distal only” was most common in the thinner group and was seen in 15 hips (Fig. 5). Cortical hypertrophy showed a significant (*p* = 0.013) difference before matching and after matching (*p* = 0.024) and was confirmed in 20% of the thicker group but not in the thinner group. Stem subsidence (≥ 3 mm) did not show a significant difference before (*p* = 0.55) or after matching (*p* = 1.0).

**Table 3:** Results of radiographic changes before and after matching

	Before matching			After matching		
	Thicker group (*n* = 39)	Thinner group (*n* = 66)	*p* value	Thicker group (*n* = 25)	Thinner group (*n* = 25)	*p* value
Stress shielding			0.015			0.26
Nothing	4	10		2	2	
1st degree	16	40		8	14	
2nd degree	11	14		9	7	
3rd degree	8	2		6	2	
Radiolucent line			< 0.0001			0.0044
Nothing	10	19		8	6	
Proximal only	11	3		3	2	
Distal only	4	39		4	15	
Proximal and distal	14	5		10	2	
Cortical hypertrophy			0.013			0.024
Nothing	29	62		20	25	
1 zone	5	3		1	0	
≥ 2 zones	5	1		4	0	
Stem subsidence			0.29			1
Nothing or < 2 mm	37	65		24	24	
≥ 3 mm	2	1		1	1

## Discussion

The most important finding of the present study was the difference in radiographic changes shortly after surgery between the two models of similar shaped stems that were both rectangular, curved, and short but had a different thickness of porous coating. “Proximal and distal” was the most common pattern of radiolucent lines in the thicker group, whereas cortical hypertrophy was also commonly observed. In contrast, “distal only” was the most common pattern of radiolucent lines for the thinner group around the reduced tip. Cortical hypertrophy was significantly less common in the thinner group. Despite the stems having similar shapes, minor differences in the design led to a difference in radiographic changes. There was no difference in terms of short-term clinical results of these two stems.

Short stem design varies greatly and there are many classifications. Feyen et al. defined standard and short stems based on stem length [27]. McTighe et al. proposed a classification based on the site of fixation and stem design [28]. Khanuja et al. classified cementless standard stems based on the style of fixation and load transfer [10]. Falez et al. proposed classification based on differences in the femoral neck resection height [29]. Ǵomez-Garćia et al. proposed to summarize existing classifications for coding [30]. As such, there are various classifications and no consensus [2]. However, the two stems used in the present study would be classified in the same group using any of these classifications: class IIIb, short stem with “standard” osteotomy in the classification by Feyen [27]; class 3A or 3B, short metaphyseal stabilized stems in the classification by McTighe [28]; type 4, shortened tapered conventional stem in the classification by Khanuja [10]; trochanter sparing in the classification by Falez [29]; and type C (cervicometaphyseal with proximal diaphyseal fixation) family, short, curved, without collar (“banana”) in the classification by Ǵomez-Garćia [30]. Despite this, the present study confirmed significant differences in radiographic changes over a short-term follow-up.

There are several reports of different radiographic changes for similar stem design. Wick et al. compared the second and third generation Zweymüller type stems, Alloclassic, and SL-Plus [31], and reported that minor differences in manufacturing, alloy materials, and morphology led to changes in the load and rotational stress on the base. There could even be different bone responses based on minor difference in elements among rectangular curved short stems that are classified in the same group. Differences in the stems used in this study include distal tip shape, sagittal taper angle, extent and thickness of porous coating, and neck surface finish (Fig. 1a, b). These differences are confirmed in the manufacturer catalogs [32, 33] or measured using enlarging digital template of ZedHip (Lexi, Toshima-ku, Tokyo, Japan), which is a three-dimensional preoperative planning tool. These differences may be associated with differences in the patterns of radiolucent lines and cortical hypertrophy where significant differences were observed between the two groups.

**Fig. 6:**
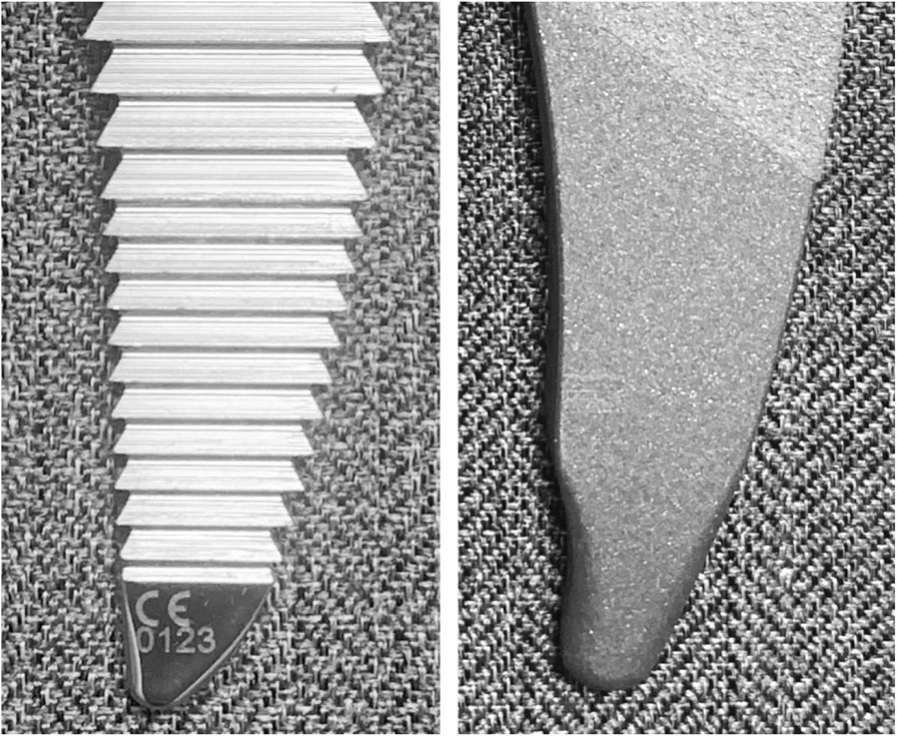
Geometrical differences in thinner group rasp (left) and actual implant of the same size (right). The actual implant showed a reduced tip but not rasp

Radiolucent lines (reactive lines) that appear and advance over a wide area around the cementless stem with movements in the stem may be a sign of loosening [19, 20, 34]. However, radiolucent lines that are about 1–2 mm in width near the Zweymüller type stem, which is a rectangular straight standard-length stem, have no impact on the clinical result and are not a sign of loosening for a distally fixed stem [11]. Zweymüller, who developed the stem, hypothesized two causes of radiolucent lines: differences in the volume between the bone resected during the stage of rasping (preparation) and the actual stem, and the difference in rigidity with the metal stem when the proximal base bone (trochanter) is in a motion caused by the muscle tension if a stem is distally fixed under the trochanter [11]. With regard to the stem of thicker group in the present study, Maier et al. reported that radiolucent lines were confirmed at 11% each in Gruen zones 1 and 7 (proximal), 2% in zone 2 and 3% in zone 6 (middle), and 2% in zone 3 and 9% in zone 5 (distal) during a follow-up of ≥ 2 years [13]. Thalmann et al. reported that radiolucent lines were confirmed at 51% in the anteroposterior image and 35% in the axial image in all zones other than the Gruen zone 2 during the first year, which decreased to 20% in the anteroposterior image and 9% in the axial image 5 years later [14]. In the present study, “proximal and distal” was the most common pattern of radiolucent lines for the thicker group (35.9% before matching and 40% after matching). On the other hand, Drosos et al. reported clinical results for the stem used in the thinner group, where radiolucent lines were confirmed at 3.3% (two hips) with distal pattern in Gruen zones 3, 4, and 5 [17]. In the present study, “distal only” was the most common pattern of radiolucent lines in the thinner group (59% before matching and 60% after matching). The reason for higher incidences compared with previous reports may be the advanced age of patients (average of 71 years prior to matching and 69 years after matching), leading to possible issues with bone quality. The tip of the stem of the thinner group was reduced, but the rasp tip was not. Radiolucent lines at the tip of the stem in the thinner group were caused by differences in the volume between the rasp and the actual stem (Fig. 6), as reported by Zweymüller [11].

**Fig. 7:**
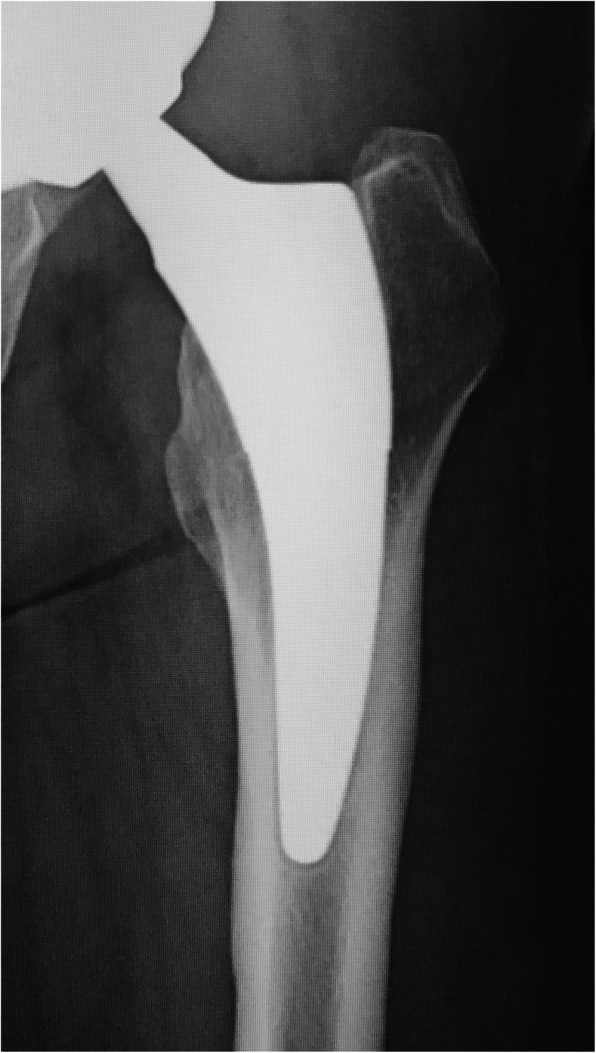
Anteroposterior radiograph at 3 years after surgery in the thicker group. Radiolucent lines were confirmed in zones 3, 4, and 5. Cortical hypertrophy was also confirmed in zones 3, 4, and 5

There are many reports on cortical hypertrophy with the stem of the thicker group. Loppini et al. performed a systematic review of nine types of short stems with different site of fixation, morphology, and neck resection height [35]. There was no report of cortical hypertrophy exceeding 10% for any stem other than the stem of the thicker group. Yan et al. evaluated changes in periprosthetic bone mineral density (BMD) in four types of short stems and two types of standard stems within 1 year of an operation in a systematic review [36]. BMD increased both inside and outside the distal area in Gruen zones 3 and 5 only for the stem of the thicker group, indicating possible distal fixation. One reason for cortical hypertrophy being common in this stem includes distal fixation [12, 13], nonideal press-fit and subsidence leading to distal two-point fixation [14], and increased lateral offset [15]. Pepke et al. argued that although fixation is proximal, the high rigidity of mediolateral bending stress could lead to cortical hypertrophy [16]. The present study included cases with coexisting distal radiolucent lines and cortical hypertrophy (Fig. 7); thus, distal fixation is not necessarily the cause of cortical hypertrophy. Regarding the stem used in the thinner group, Drosos et al. observed 6.6% cortical hypertrophy in a year-long observation of 61 hips in the thinner group [17]. In the present study, 25.6% cortical hypertrophy was confirmed before matching and 20% after matching in the thicker group. In contrast, 6.1% was confirmed before matching and 0% after matching in the thinner group. These stems have similar shape and use the same material (titanium alloy); thus, the rigidity of the stem itself was assumed to be similar.

Differences in the thickness of the porous coating of these stems may lead to differences in the patterns of the radiolucent line and cortical hypertrophy. If stems have the same shape, the thicker coating requires more impaction stress to reach the same depth [37]. Stems with a thicker coating require more impaction stress for appropriate installation, and the press-fit force is focused on the distal part of the coating (around the middle part of the stem). Thus, the load stress is focused on the middle part of the stem, and micromotions are generated in the proximal and distal parts if the stem is only fixed at the middle part due to differences in the rigidity between the stem and bone [11]. This may explain why “proximal and distal” is the most common pattern of radiolucent lines in the thicker group. As the stem comes into contact with the cortex in Gruen zones 3 and 5 (distal part), the stress leads to increase in cortical hypertrophy. However, since standard stems with good long-term results also present various radiographic changes [38], it remains unclear how such imaging findings in rectangular curved short stems change over a long period of time, impacting the clinical results. There have been reports that radiographic changes may be linked to early revision [19, 20]; thus, although the short-term clinical results showed no difference in the present study, these radiographic changes would be of clinical concern.

The present study has several limitations. First, acetabular components and bearing couples were not uniform, even after matching. In the present cases, we used BIOLOX delta (CERAMTEC GmbH, Plochingen, Germany) as the femoral head, but the diameter of the femoral head varied between φ28 and φ40 mm. Two types of acetabular component and liner were used in the thicker group, whereas four types were used in the thinner group. However, in terms of differences in the material of the bearing couples and the femoral head diameter, friction can be ignored for the short period (within 5 years) that we examined in the present study [39–42]. Second, we did not make a comparison of patient-reported outcome measures (PROMs) [43]. While JOA hip score is not a PROM, many reports have argued that stress shielding, radiolucent line, and cortical hypertrophy, which were imaged in the present study, are unrelated to clinical symptoms [5, 6, 11–15, 17, 24]. Furthermore, the follow-up period was short and the number of cases was small. It is important to observe how the changes seen in the present study would change over longer follow-up periods. One of the strengths of the present study was that it used the same approach performed by the same surgeon. Furthermore, a propensity score-matched analysis was performed and comparisons were made after eliminating demographic differences, including femoral canal morphology.

## Conclusions

Two similarly shaped rectangular curved short stems exhibited significant differences in radiographical changes over a short period of time compared with after equalizing demographic data including the femoral canal morphology through a propensity score-matched analysis. Even when the morphology is similar, minor differences in stem design, such as thickness of the porous coating, can lead to radiographic changes shortly after surgery. The most common pattern of radiolucent lines in the thicker group was proximal and distal, while it distal only was most common in the thinner group. The incidence of cortical hypertrophy was significantly higher in the thicker group, whereas the result was more “silent” for the thinner group.

## Data Availability

The datasets used and/or analyzed during the current study are available from the corresponding author on reasonable request.

## Abbreviations

BMD: Bone mineral density
JOA: Japanese Orthopaedic Association
PROM: Patient-reported outcome measures
THA: Total hip arthroplasty
